# William W. Fouché, D.D.S.

**Published:** 1891-01

**Authors:** 


### Obituary.

### WILLIAM W. FOUCHÉ, D.D.S.

Dr. William W. Fouché died in Philadelphia, November 13, 1890, of gastritis. He had reached his seventy-seventh year, retaining a remarkable activity until the close of his life.

Dr. Fouché was born in New York City, September 29, 1814. He obtained his first instruction in dentistry in the office of Dr. Samuel S. Fitch, of that city.

In 1834 he located in Philadelphia, and commenced, in this city, the practice of his chosen profession, receiving the honorary degree of Doctor of Dental Surgery from the Philadelphia College of Dental Surgery at the close of its first session, in the spring of 1853.

Dr. Fouché was one of the promoters and early members of the Pennsylvania Association of Dental Surgeons, which was organized in 1845. The younger members who are now living will remember the interest he took in the discussion of subjects before that body. In 1852 he was elected its president.

For many years Dr. Fouché was a member of the Board of Corporators of the Pennsylvania College of Dental Surgery. Of this Board he was secretary during most of the years of his connection with it.

On July 5, 1838, he married Miss Mary Wilson, who, with two sons and two daughters, survives him.

The commercial talent, which is so rare with professional men, was possessed by Dr. Fouché in an eminent degree, and, as a result of this, he left a large estate for the benefit of the surviving members of his family. P.

